# Pep12 is important for proteasome microautophagy under low glucose conditions

**DOI:** 10.17912/micropub.biology.001579

**Published:** 2025-05-07

**Authors:** Kyle VanderVen, Conner Butcher, Remi' Fokine, Jianhui Li

**Affiliations:** 1 Biomedical Engineering and Science, Florida Institute of Technology, Melbourne, Florida, United States

## Abstract

Autophagic degradation of proteasomes is a highly conserved mechanism for regulating proteasome homeostasis in eukaryotes. Here we show that Pep12, a t-SNARE protein, is important for intralumenal vesicle formation in the vacuole and proteasome microautophagy under low glucose conditions. Deleting *PEP12* in yeast cells, *Saccharomyces cerevisiae* , blocked proteasome fragmentation, by which the ESCRT-dependent microautophagy selectively degrades aberrant proteasomes. Accumulation of aberrant proteasomes interfered with proteasome condensate formation in *pep12∆* cells. Autophagic bodies were present, but intralumenal vesicles and proteasome microautophagy were absent in the vacuole of *pep12∆* cells. Therefore, Pep12 plays an important role in proteasome microautophagy.

**Figure 1. Pep12 is important for ILV formation and proteasome microautophagy under low glucose conditions:**
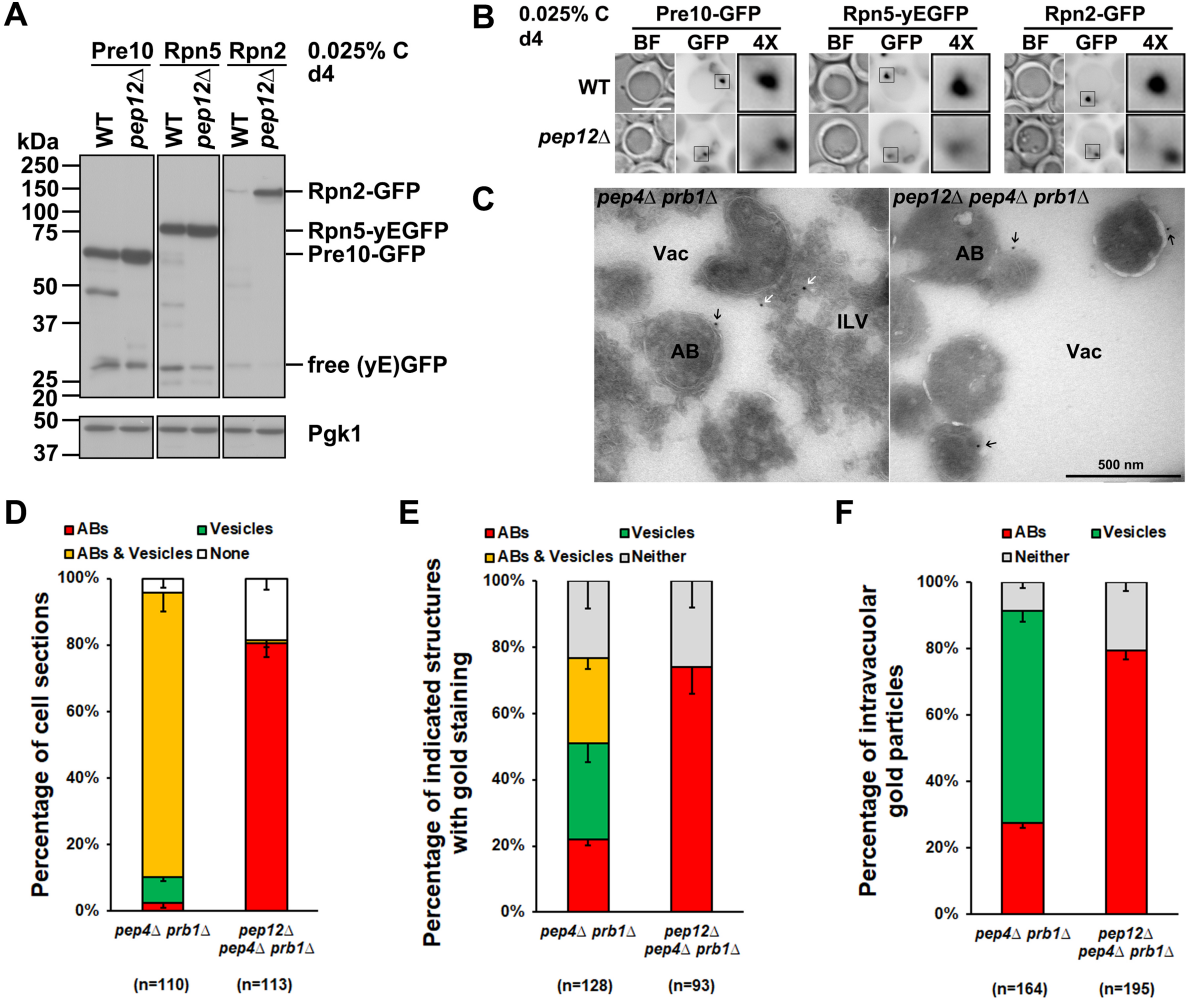
**(A)** Anti-GFP immunoblot analyses of Pre10-GFP, Rpn5-yEGFP, and Rpn2-GFP in WT and *pep12∆* cells. Pgk1 serves as a loading control. Representative blots from at least three experiments are shown. **(B)** Fluorescence images of Pre10-GFP, Rpn5-yEGFP, and Rpn2-GFP in WT and *pep12∆* cells. Proteasome puncta were observed in *pep12∆* cells. 4x, 4-fold enlargement of the boxed regions. BF, bright field. Scale bar: 5 µm. Representative images from at least three experiments are shown. ( **C)** Cryo-immunogold electron micrographs of proteasomes in *pep4∆ prb1∆* and *pep12∆* *pep4∆ prb1∆* cells. Sections were immunolabeled with anti-CP primary antibody and gold-conjugated Protein A (black arrows indicate AB-associated proteasomes and white arrows indicate ILV-associated proteasomes). Vac, vacuole; ILV, intralumenal vesicle; AB, autophagic body. Representative images are shown. **(D)** Percentage of cell sections with the indicated intravacuolar structures (ABs; vesicles; ABs and vesicles; none) in the strains used for panel C. **(E)** Percentage of the indicated structures with gold particle staining in the cells used for panel C. **(F)** Percentage of intravacuolar gold particles staining either ABs or vesicles or neither in the cells used for panel C. ‘n’ represents cell sections counted in panels D and E, and intravacuolar gold particles counted in panel F. Results plotted as mean±s.d. Cells were harvested from cultures grown in SC medium containing 0.025% glucose for four days.

## Description

The ubiquitin-proteasome system and autophagy are two major cellular protein degradation systems that maintain cell health and increase cell fitness under stress conditions (Pohl and Dikic, 2019). The 26S proteasome is a large multiprotein complex and comprises the core particle (CP) and the regulatory particle (RP). The RP consists of the lid and the base subcomplexes (Bard et al., 2018). Proteasomes selectively degrade cellular proteins to maintain protein homeostasis in cell health (Rousseau and Bertolotti, 2018). Autophagy is a self-degradative process, recycling cellular components for cell survival, especially under stress conditions (Reggiori and Klionsky, 2013). Autophagic degradation of proteasomes is a highly conserved pathway and has been identified in yeast, plant, and mammalian cells (Cohen-Kaplan et al., 2016; Marshall et al., 2015; Nemec et al., 2017; Waite et al., 2016). In budding yeast cells, *Saccharomyces cerevisiae* , proteasome macroautophagy and microautophagy have been identified under low glucose conditions (Li et al., 2019; Li and Hochstrasser, 2020). The endosomal sorting complex required for transport (ESCRT)-dependent microautophagy of proteasomes selectively degrades aberrant proteasomes during reversible proteasome condensate formation (Li and Hochstrasser, 2022). Meanwhile, AMP-activated protein kinase (AMPK), a master cellular energy regulator, regulates the ESCRT-dependent microautophagy of proteasomes (Li et al., 2019). Besides ESCRTs and AMPK, the cellular components required for proteasome microautophagy are poorly understood. Here, we have identified that Pep12, a target membrane receptor (t-SNARE (soluble N-ethylmaleimide-sensitive-factor attachment protein receptor)), is important for the proteasome microautophagy pathway.

To examine proteasome trafficking and autophagic degradation under low glucose (0.025% C as indicated in figure panels) conditions, we used yeast genetics approaches to generate yeast strains expressing GFP-tagged proteasome subunits, a CP subunit Pre10 (Pre10-GFP), a lid subunit Rpn5 (Rpn5-yEGFP), and a base subunit Rpn2 (Rpn2-GFP), respectively, in wild type (WT) and *pep12∆* cells. The resulting yeast strains were glucose-starved since this is a well-established condition to induce proteasome autophagy and cytoplasmic proteasome condensate formation simultaneously (Butcher et al., 2025; Li et al., 2019). We used the “free GFP release” method to examine proteasome autophagy (Torggler et al., 2017). Proteasome-GFP is transported to the vacuole (lysosome in mammalian cells) by autophagy. Then, the proteasome is degraded, while GFP is more resistant to vacuolar hydrolases, accumulating free GFP in the vacuole. Therefore, proteasome autophagy can be monitored by immunoblotting with GFP antibodies. Proteasome fragmentation, the partial proteasome degradation bands between the full-length GFP fusion proteasome subunit and the free GFP ( Figure 1A ), has been identified as a readout for proteasome microautophagy, while the free GFP level reflects proteasome macroautophagy (Li et al., 2019; Li and Hochstrasser, 2022). Proteasome macroautophagy and microautophagy are induced simultaneously in yeast cells under low glucose conditions (Li and Hochstrasser, 2020). The free GFP levels from RP subunits Rpn5 and Rpn2 were reduced, while those from the CP subunit Pre10 were normal in *pep12∆* compared to WT cells ( Figure 1A ). This result indicates Pep12 is involved in RP but not CP macroautophagy. Therefore, CP and RP subcomplexes are degraded through different pathways under low glucose. This has also been observed under nitrogen starvation conditions, where Ubp3 is involved in CP but not RP autophagy (Waite et al., 2016). Proteasome fragmentation of Pre10, Rpn5, and Rpn2 was blocked in *pep12∆* compared to WT cells ( Figure 1A ), suggesting that proteasome microautophagy is interrupted, thereby accumulating aberrant proteasomes in the cells. The defects in proteasome microautophagy can subsequently interfere with reversible proteasome condensate formation, as observed in the ESCRT deletion mutants (Li and Hochstrasser, 2022). Therefore, we used fluorescence microscopy to examine proteasome condensates in *pep12∆* cells. Condensed and bright proteasome condensates were observed in WT cells. In contrast, diffused and dispersed proteasome signals were observed near the vacuolar membrane in *pep12∆* cells ( Figure 1B, highlighted with squares in the images). This result indicates a defect in proteasome condensate formation and proteasome trafficking to the vacuole lumen for microautophagic degradation.

To confirm the importance of Pep12 in proteasome microautophagy, we used immunogold labeling electron microscopy (EM) to label proteasome CPs with 10 nm gold beads ( Figure 1C, pointed by the arrows). There are two major proteases in the vacuole, encoded by the *PEP4* and *PRB1* genes in yeast cells (Hecht et al., 2014). We deleted both genes to retain the autophagic body (AB) and intralumenal vesicle (ILV) structures in the vacuole. In line with our previous studies (Li et al., 2019), about 85% of cell sections had both ABs and ILVs in the vacuole in *pep4∆ prb1∆* cells. Interestingly, about 81% of cell sections had ABs, while less than 1% had ILVs in *pep12∆ pep4∆ prb1∆* cells ( Figure 1D ). The results showed that Pep12 deletion caused a general deficiency of ILV formation and subsequent proteasome microautophagy, but did not affect AB formation in macroautophagy under low glucose conditions ( Figure 1C and 1D). We further quantified ABs and ILVs with proteasome CP staining and found that about 75% of cell sections had proteasome staining with ABs in *pep12∆ pep4∆ prb1∆* cells, but distributed roughly evenly between ABs and ILVs in *pep4∆ prb1∆* cells ( Figure 1E ). We also quantified the distribution of gold beads in the vacuole and found that about 79% of gold beads had stained ABs in *pep12∆ pep4∆ prb1∆* cells. About 27% and 64% had stained ABs and ILVs in *pep4∆ prb1∆* cells ( Figure 1F ). Our results demonstrate that Pep12 is important for the proteasome microautophagy pathway.

SNAREs are a family of proteins critical for membrane fusion and are categorized as target membrane t-SNAREs or vesicle membrane v-SNAREs (Grissom et al., 2020). Pep12 is a multifunctional yeast syntaxin protein with a C-terminal hydrophobic region. It functions as t-SNARE on the prevacuolar compartment endosome for vesicle trafficking between the Golgi and the endosome/vacuole (Becherer et al., 1996). Pep12 is necessary for protein trafficking to the vacuole (Gerrard et al., 2000) and can also be degraded in the vacuole (Kawamata et al., 2022). Pep12 is important for phagophore closure during autophagosome formation under certain conditions, such as nitrogen starvation (Zhou et al., 2017). However, Pep12 is unnecessary for autophagosome formation under low glucose conditions, since ABs were observed in *pep12∆ pep4∆ prb1∆* cells ( Figure 1C ). Therefore, Pep12 may play different roles in autophagy under specific conditions. The ESCRTs are essential for ILV formation under low glucose conditions (Li et al., 2019; Li and Hochstrasser, 2022). Moreover, Pep12 is important for the ESCRT-dependent ILV formation in vacuole and proteasome microautophagy, but how Pep12 is involved in this process still needs further investigation.

## Methods

**Yeast strains and cell growth**

Yeast strains used in this study are listed in the reagents section. Yeast cells were grown overnight at 30°C with vigorous agitation in synthetic complete (SC) medium (Li and Hochstrasser, 2022). Cells were back-diluted in SC medium and grown to mid-log phase. The mid-log cells were harvested and rinsed once with sterile ultrapure water. The cells were then resuspended in SC medium containing 0.025% glucose and incubated at 30°C for four days.

**Protein extraction and Western blotting**

Total proteins were extracted using the Kushnirov method (Kushnirov, 2000), and Western blotting was performed as described previously with minor modifications (Li et al., 2016). The equivalent of one optical density unit at 600 nm (OD _600_ ) of cells were harvested, subjected to 0.1M NaOH treatment, and then boiled in SDS sample buffer (10% glycerol, 2% SDS, 0.1 M DTT, 62.5 mM Tris-HCl pH 6.8, 4% 2-mercaptoethanol, 0.008% Bromophenol Blue).

Equal volumes of the protein extracts were loaded onto 10% (v/v) SDS-PAGE gels, followed by the transfer of proteins to PVDF membranes (EMD Millipore, catalog #IPVH00010). The membranes were incubated with JL-8 anti-GFP monoclonal antibody (TaKaRa, catalog #632381) at a 1:2000 dilution and anti-Pgk1 monoclonal antibody (Abcam, catalog #ab113687) at a 1:10,000 dilution. Primary antibody binding was followed by anti-mouse-IgG (GE Healthcare, catalog #NXA931V) secondary antibody conjugated to horseradish peroxidase at a 1:10,000 dilution. The membranes were incubated in ECL detection reagent (Mruk and Cheng, 2011). The ECL signals were detected using film (Thomas Scientific, catalog #1141J52).

**Fluorescence microscopy**

Yeast cells were visualized on an Axioskop microscope (Carl Zeiss) equipped with a Plan-Apochromat 100×/1.40 NA oil DIC objective lens, a CCD camera (AxioCam MRm; Carl Zeiss), and an HBO100W/2 light source. Images were taken using AxioVision software with an auto-exposure setup and processed using Adobe Photoshop CC software.

**Immunogold labeling electron microscopy**

Immunogold labeling EM was performed as described previously (Li et al., 2019). Briefly, yeast cells were fixed with 4% paraformaldehyde (PFA) and resuspended in 10% gelatin. The solidified blocks were frozen in liquid nitrogen and cut into 60 nm thickness sections on a Leica Cryo-EM UC6 UltraCut. The cell sections were placed on carbon/Formvar-coated grids and floated in a dish of PBS for immunolabeling. Grids were blocked on 1% fish skin gelatin in PBS and incubated with a primary rabbit anti-20S antibody (Enzo Life Sciences, catalog #BML-PW9355) at a 1:200 dilution. Protein A-gold beads (10 nm; Utrecht Medical Center) were used for secondary staining. The grids were rinsed in PBS, fixed with 1% glutaraldehyde, rinsed again, and stained with a mixture of 0.5% uranyl acetate to methylcellulose. Grids were viewed under a transmission electron microscope (FEI Tecnai G2 Spirit BioTWIN) at 80 kV. Images were taken using a SIS Morada 11-megapixel CCD camera and iTEM (Olympus) software. Acquired images were processed using Adobe Photoshop CC software.

## Reagents

**Table:** 

**Strain**	**Genotype**	**Reference**
JLY398	*MATa his3-∆200 leu2-3, 112 ura3-52 lys2-801 trp1-1 PRE10-GFP::HIS3*	(Li et al., 2019)
JLY1783	*MATa/α his3-∆200 leu2-3, 112 ura3-52 lys2-801 trp1-1 pep12∆::hphMX PRE10-GFP::HIS3*	This study
JLY2366	*MATa his3-∆200 leu2-3, 112 ura3-52 lys2-801 trp1-1 RPN5-yEGFP::HIS3*	This study
JLY1776	*MATa/α his3-∆200 leu2-3, 112 ura3-52 lys2-801 trp1-1 pep12∆::hphMX RPN5-yEGFP::HIS3*	This study
JLY272	*MATa his3-∆200 leu2-3, 112 ura3-52 lys2-801 trp1-1 RPN2-GFP::HIS3*	(Li et al., 2019)
JLY1785	*MATa/α his3-∆200 leu2-3, 112 ura3-52 lys2-801 trp1-1 pep12∆::hphMX RPN2-GFP::HIS3*	This study
MHY7796	*MATa his3-∆200 leu2-3, 112 ura3-52 lys2-801 trp1-1 pep4∆::natMX prb1∆::kanMX*	(Li et al., 2019)
JLY1847	*MATa/α his3-∆200 leu2-3, 112 ura3-52 lys2-801 trp1-1 pep12∆::hphMX pep4∆::natMX prb1∆::kanMX*	This study
